# Supplementary material for: Substrate accessibility regulation of human TopIIa decatenation by cohesin
Source: Nat Commun. 2025 Aug 5;16:7200. doi: 10.1038/s41467-025-62505-3 (PMC12326010; doi:10.1038/s41467-025-62505-3)

Supplementary Information

Supplementary Information for:

Substrate accessibility regulation of human TopIIa decatenation by cohesin

Corresponding to:

Luis Aragon (luis.aragon@lms.mrc.ac.uk), DNA Motors Group, MRC Laboratory of Medical Sciences, Du Cane Road, London W12 0HS, UK.

David S. Rueda (david.rueda@imperial.ac.uk), Single Molecule Imaging Group, MRC Laboratory of Medical Sciences, Du Cane Road, London W12 0HS, UK

This file includes Supplementary Figures 1-5 and original gels.

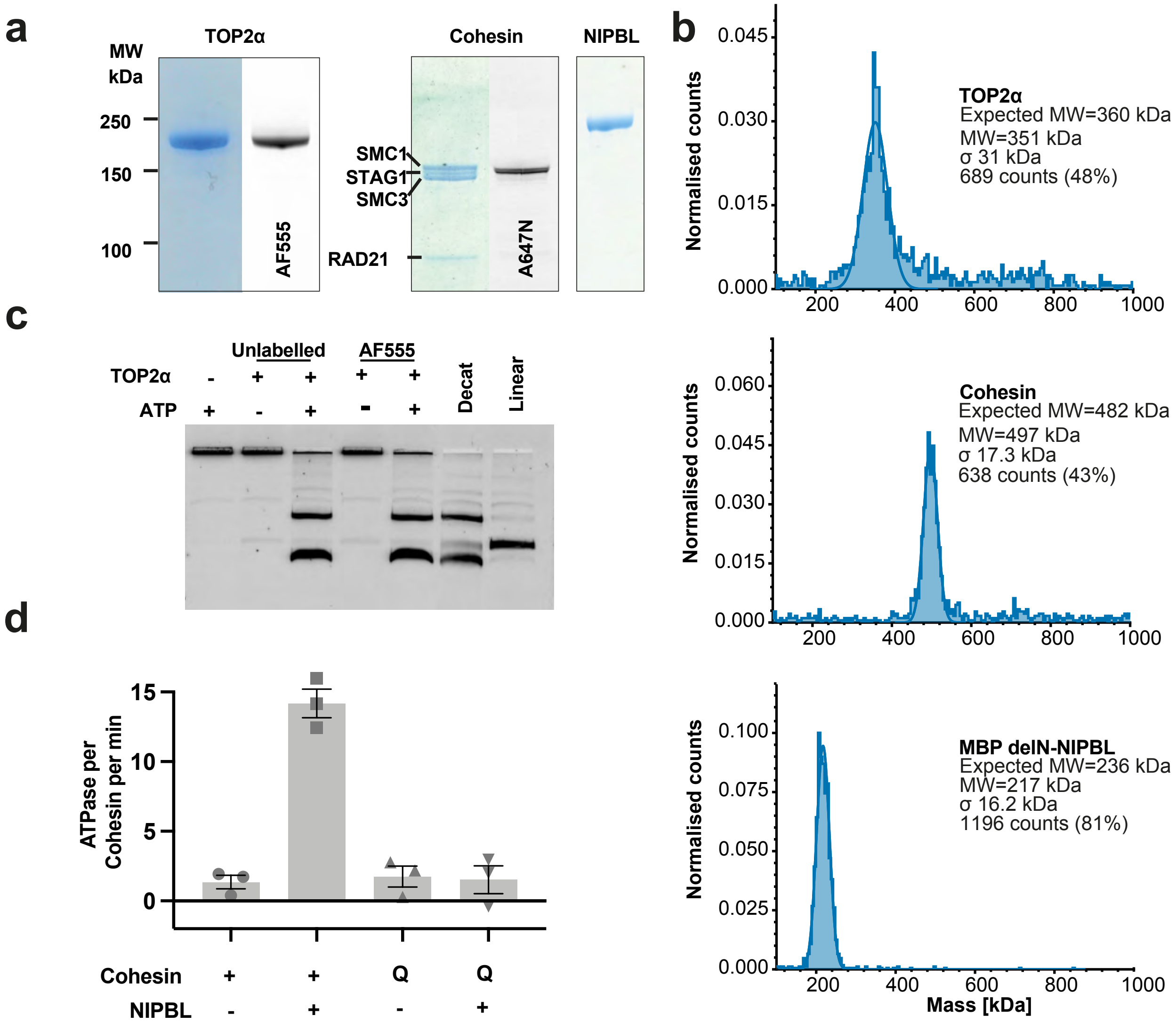

Supplementary Figure 1: Purified protein used in this study. a, SDS page analysis of protein samples used in this study, stained either with Coomassie or imaged via conjugated fluorophore. b, Mass-photometry analysis of proteins used in this study, indicating expected and measured molecular mass. c, Decatenation assay, demonstrating ATP dependent decatenation of kinetoplast DNA (kDNA) by unlabelled and labelled TOP2α. Decatenated control (Decat) and cleaved linear kDNA are loaded to demonstrate DNA is being decatenated rather than cleaved. d, ATP hydrolysis assay of cohesin samples in the presence of 20-fold excess of 50 bp DNA. Q indicates ATPase hydrolysis deficient mutation in the Q-loop of ATPase site. N=3, error bars indicate one standard error.

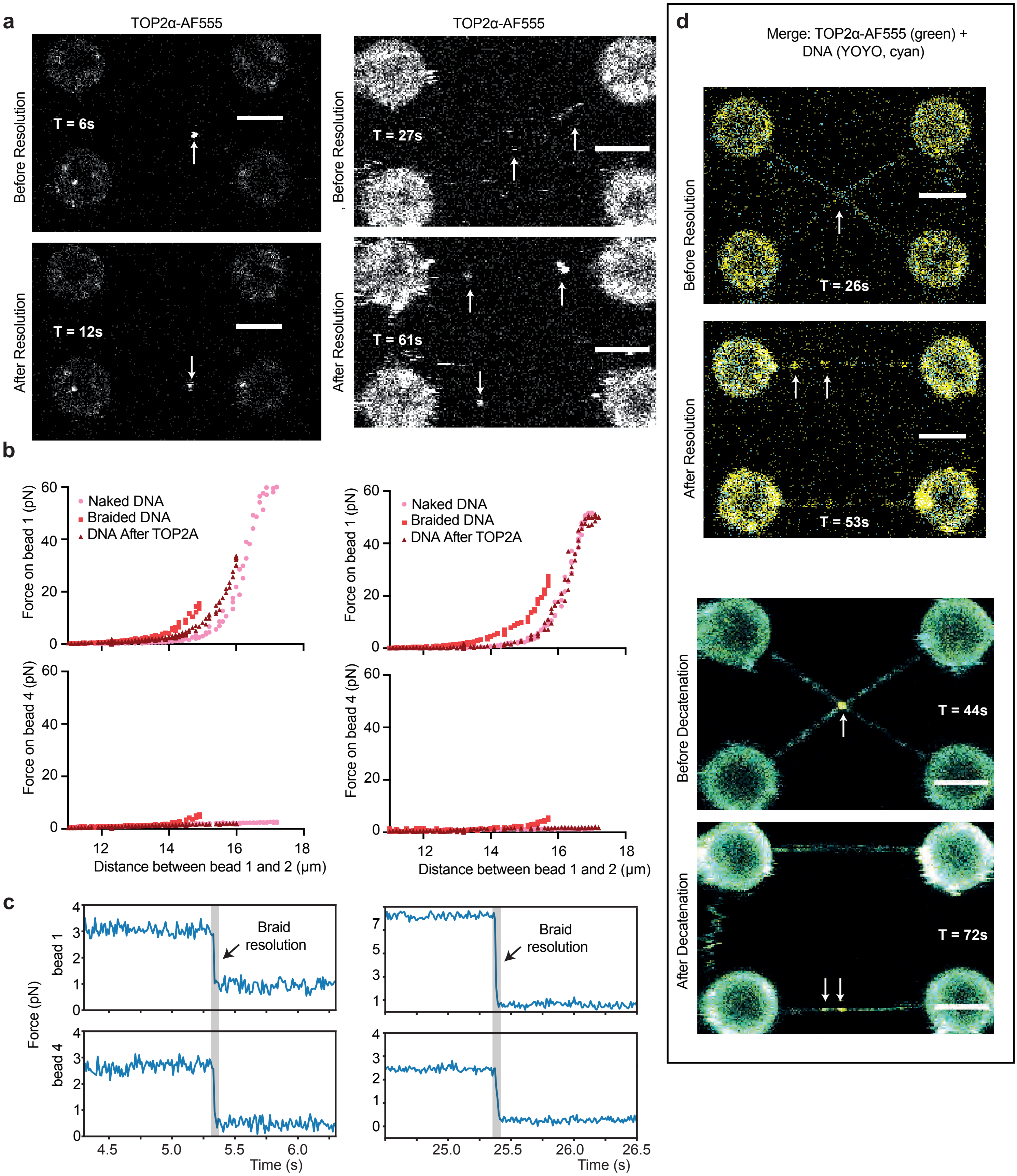

Supplementary Figure 2: Additional examples of single-molecule visualisation of TOP2α DNA braid resolution. a, Examples of resolution events imaged with TOP2α-AF555 in the absence of YOYO. b and c, Force extension curves of DNA substrate and force vs time curve of a resolution event corresponding to examples in a, respectively. d, Two additional examples of braid resolution events imaged with YOYO and TOP2α-AF555. White arrows indicated TOP2α-AF555 binding, scalebar is 4 μm.

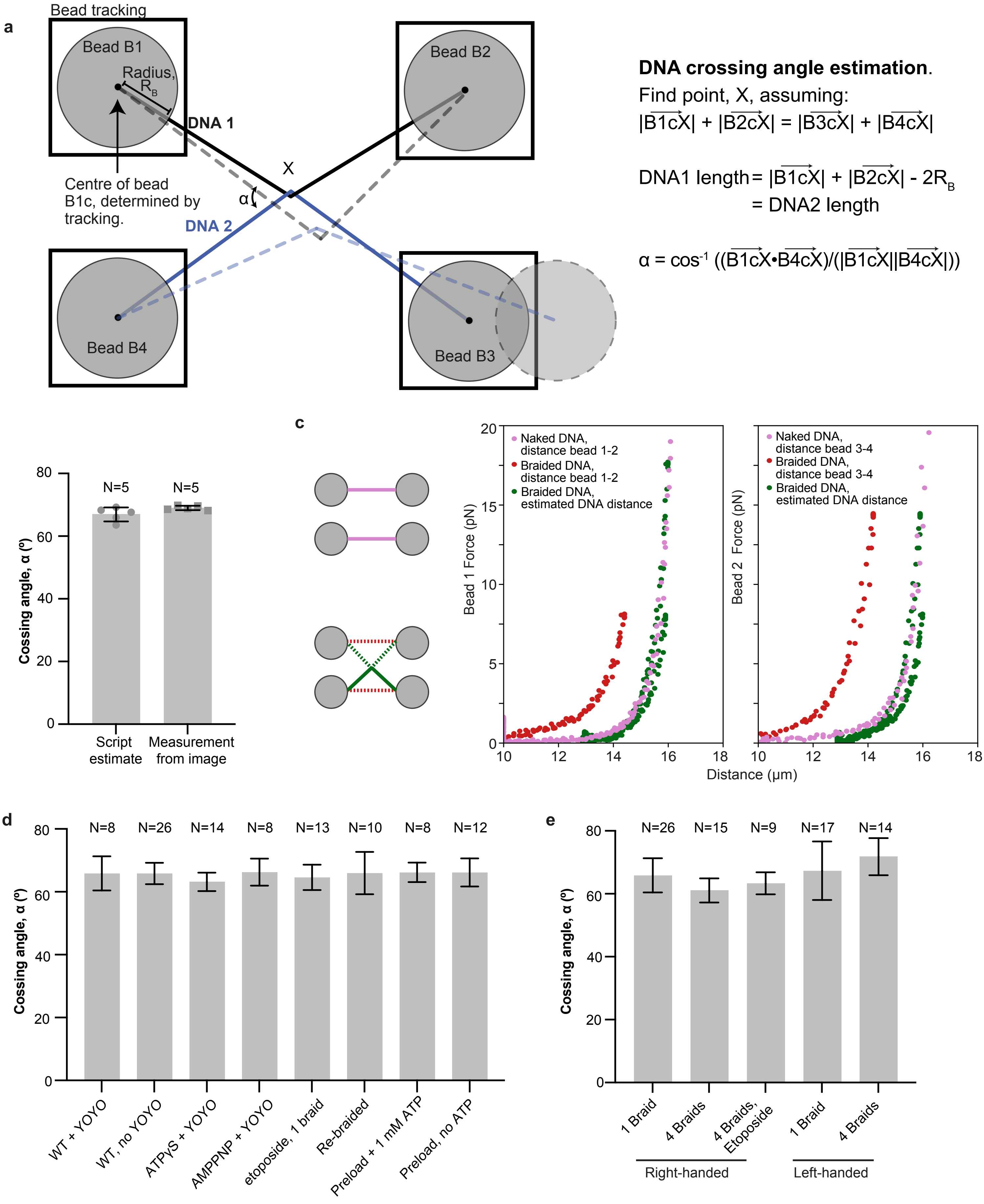

Supplementary Figure 3: Braided DNA crossing angle and geometry analysis. a, A schematic of braided DNA indicating the relationship between the bead positions, the DNA length, the angle between the two DNAs and the assumptions used to estimate DNA length and crossing angle. If one DNA is stretched by moving one bead, as is done during force extension measurements shown in Fig. 1f and Supplementary Fig. 2b, the geometry will be altered to maintain equal DNA lengths, as shown in dashed lines. b, Comparison of crossing angle estimate with crossing angle measured directly form YOYO-stained DNA. Source data are provided as a Source Data file c, Estimate of DNA length from bead position corrects force extension curve from appearing to have a shorter contour length, red, towards the length expected for lambda DNA, green, where naked lambda DNA is shown in pink. d and e, Analysis of mean crossing angle of DNA in data shown in Fig. 3a and 4b, respectively. In all cases error bars indicated standard error, and sample size, N, is illustrated. Source data are provided as a Source Data file.

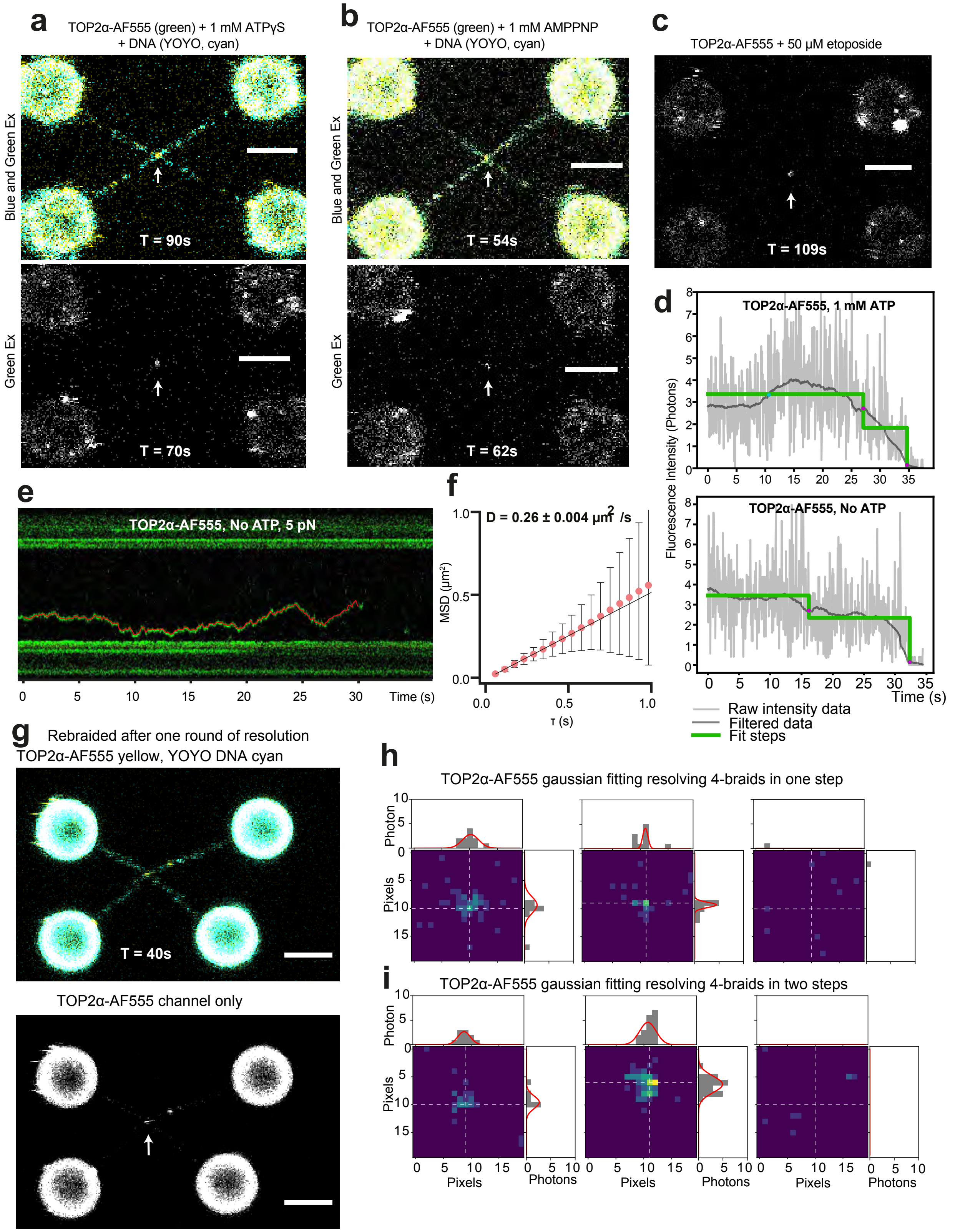

Supplementary Figure 4: TOP2α ATP hydrolysis and DNA association. a, Example image of TOP2α-AF555 in the presence of 1 mM ATPγS, with blue and green excitation and only green excitation. b, Example image of TOP2α-AF555 in the presence of 1 mM AMPPNP, with blue and green excitation and only green excitation. c, Example image of TOP2α-AF555 failing to resolve a DNA braid in the presence of 50 μM etoposide. d, Fluorescence intensity vs time of TOP2α-AF555 diffusing on DNA in the presence and absence of 1 mM ATP, with two steps fit to the loss of photon intensity. e, Example tracking of a TOP2α-AF555 diffusing on DNA, used to calculate the mean squared displacement (MSD) in f. Error bars in f, indicate variance of MSD. g, Example image of TOP2α-AF555 failing to resolve DNA after being re-braided following one round of successful resolution. Left image shows a merge of both TOP2α-AF555 and YOYO imaging channels and right shows TOP2α-AF555 channel alone. h, Intensity Gaussian fitting of signal in Fig. 4d. i, Intensity Gaussian fitting of signal in Fig. 4e. In all cases, scalebar is 4 μm.

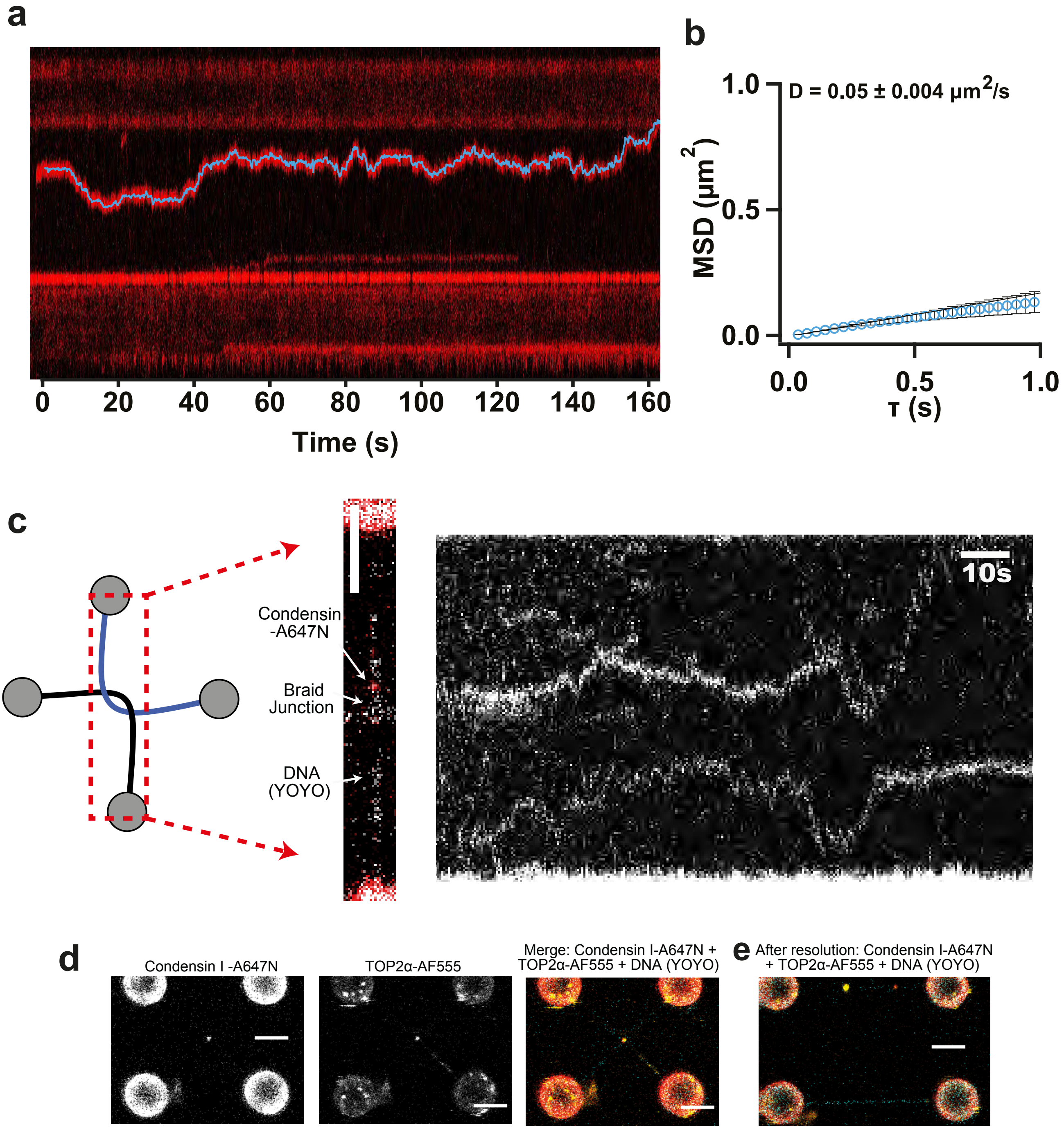

Supplementary Figure 5: Cohesin DNA diffusions and condensin I interaction with braided DNA. a, Example tracking of Cohesin-A647N /MBP-ΔN-NIPBL diffusing on DNA, used to calculate the mean squared displacement (MSD) in b. Error bars in b, indicate variance of MSD. c, Condensin I-A647N kymograph on braided DNA substrate. Kymograph is generated by scanning a limited area of reorientated beads and collapsing each scan into one line. This example shows condensin I-A647N diffusing into the junction and failing to stably associate. d, Example of Condensin I-A647N colocalising with TOP2α-AF555 at a braid junction and allowing braid resolution, e. In all cases, scalebar is 4 μm.

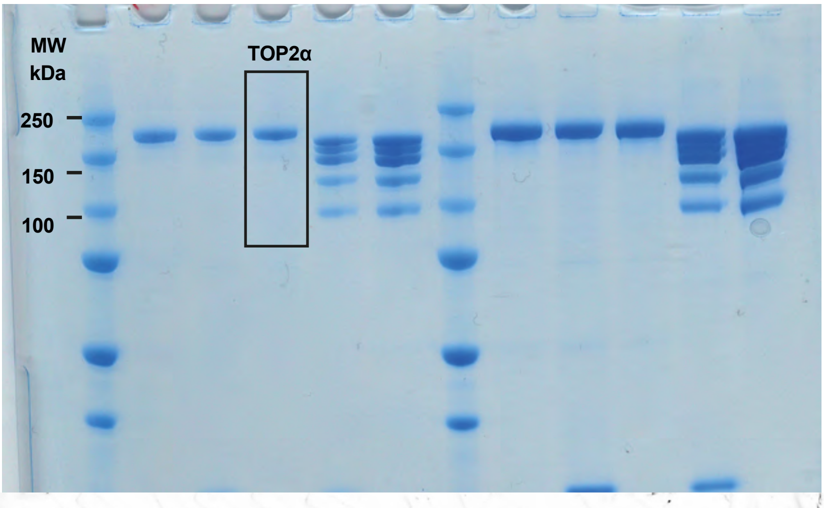
MW
kDa
250
150
100
TOP2α

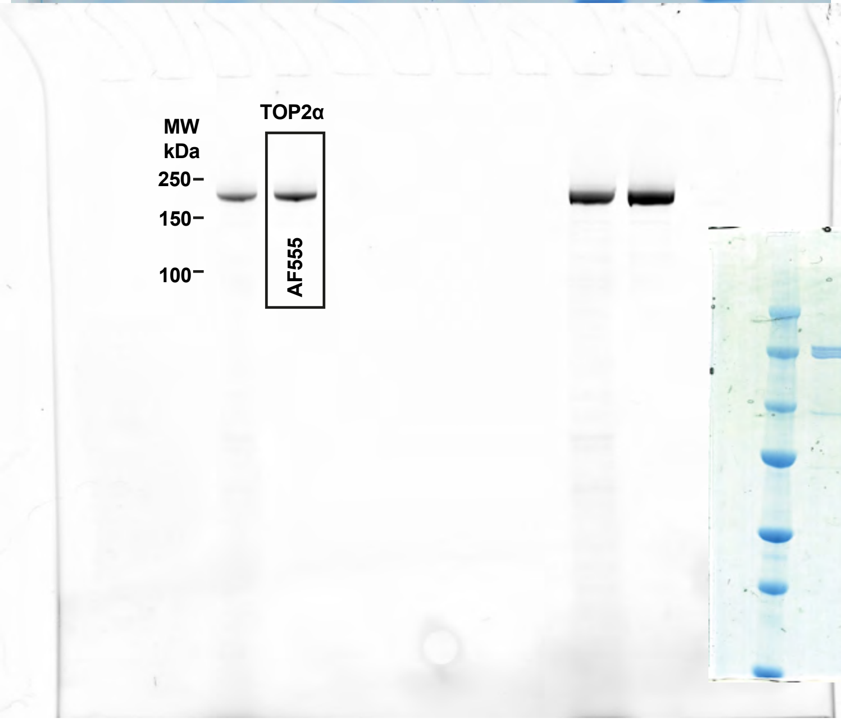
MW
kDa
250
150
100
TOP2α
AF555

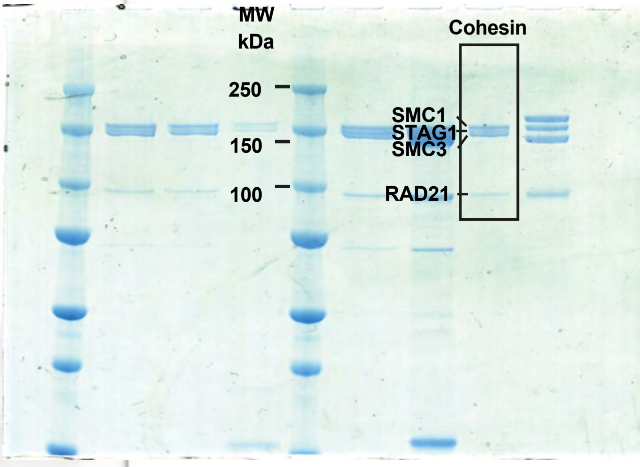
MW
kDa
250
150
100
Cohesin
SMC1
STAG1
SMC3
RAD21

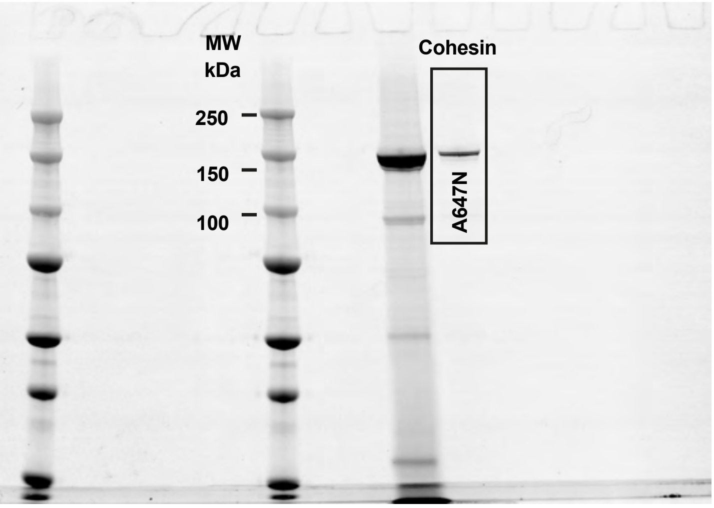
MW
kDa
250
150
100
Cohesin
A647N

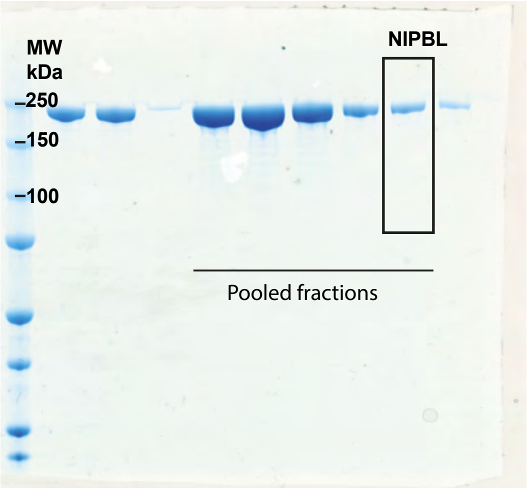
MW
kDa
250
150
100
NIPBL
Pooled fractions

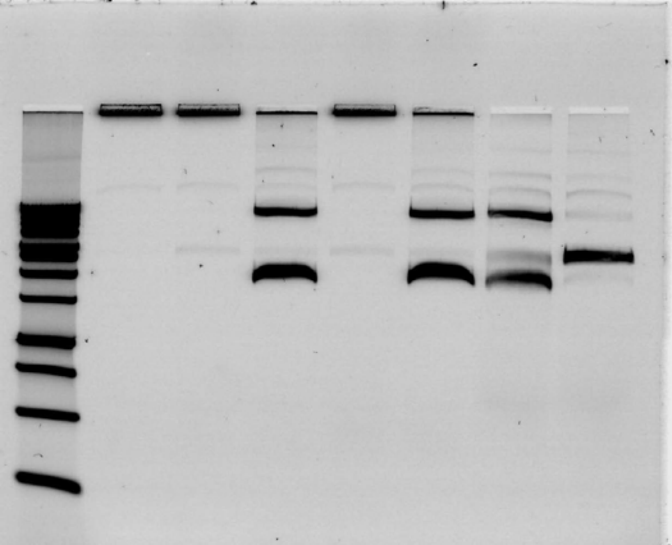

Supplement: Supplementary file 1 — Supplementary Information [file 41467_2025_62505_MOESM1_ESM.pdf]
