# Supplementary material for: Substrate accessibility regulation of human TopIIa decatenation by cohesin
Source: Nat Commun. 2025 Aug 5;16:7200. doi: 10.1038/s41467-025-62505-3 (PMC12326010; doi:10.1038/s41467-025-62505-3)
Supplement: Supplementary file 2 — Description of Additional Supplementary Files [file 41467_2025_62505_MOESM2_ESM.pdf]

Description of Additional Supplementary Files

Supplementary Movie 1: Optical tweezer generation of braided DNA substrate

Supplementary Movie 2: Real time visualisation of DNA braid resolution by TOP2α

Supplementary Movie 3: Cohesin form DNA bridges that are resolved by SDS

Supplementary Movie 4: Cohesin prevents TOP2α braid resolution
